# Advances in p53 Therapies for Oral Squamous Cell Carcinoma: A Literature Review

**DOI:** 10.3390/cimb48101013

**Published:** 2026-10-01

**Authors:** Victor Manuel Martínez Aguilar, Cristina Trejo-Solís, Roxana Magaña-Maldonado, Keneth Emmanuel Chac-Couoh, Viviana Pitones-Rubio, Juan Carlos Gallardo-Pérez, Bertha Arelly Carrillo Ávila, Angélica Hurtado-Camarena, Elda Georgina Chávez-Cortéz

**Affiliations:** 1 Faculty of Dentistry, Autonomous University of Yucatan, Merida 97000, Yucatan, Mexico; victor.martinez@correo.uady.mx (V.M.M.A.); a13004@alumnos.uady.mx (K.E.C.-C.); arelly.carrillo@correo.uady.mx (B.A.C.Á.); 2 Experimental Laboratory for Neurodegenerative Diseases and Cell Reprogramming Laboratory, National Institute of Neurology and Neurosurgery, Mexico City 14269, Mexico; cristina.trejo@innn.edu.mx (C.T.-S.); rmagana@innn.edu.mx (R.M.-M.); 3 Faculty of Dentistry, Autonomous University of Baja California, Mexicali 21040, Baja California, Mexico; viviana.pitones@uabc.edu.mx; 4 Department of Cardio-Renal Pathophysiology, Department of Biochemistry, National Institute of Cardiology, Mexico City 14080, Mexico; jcgallardo@ciencias.unam.mx; 5 Faculty of Sciences, National Autonomous University of Mexico, Mexico City 04510, Mexico

**Keywords:** cell-cycle, mouth neoplasms, oral squamous cell carcinoma, p53 therapies, *TP53*, Tumor Suppressor Protein p53

## Abstract

p53 is a tumor suppressor involved in cell-cycle regulation and DNA repair. Studies have shown that altered p53 expression in oral squamous cell carcinoma (OSCC) is associated with a poor prognosis and increased tumor aggressiveness, suggesting it could be considered a potential prognostic marker. A search was performed using keywords such as oral squamous cell carcinoma, *TP53*, and p53 therapies in the PubMed and Web of Science databases, with a timeframe from 2020 to 2025. In this review, we examined various therapies targeting p53 that have been proposed in recent years. These therapies have been tested in vitro, in vivo, and in some cases, through clinical trials. Their goal is to restore the conformation and activity of mutated p53 by identifying small molecules that restore p53 function and eliminate mutated cells. Although some of these therapies show promising results, further clinical studies are needed to assess their efficacy and safety in OSCC patients.

## 1. Introduction

Oral cancer is one of the most frequently reported diseases worldwide and is ranked as the 17th most common cause of death globally [1]. OSCC is the most prevalent form of head and neck squamous cell carcinoma (HNSCC) [2,3], and represents approximately 90% of all cases [4]. Furthermore, OSCC poses a significant global health burden, with an estimated 389,846 new cases and 188,438 deaths reported annually, according to the 2022 estimates from the Global Cancer Observatory [5].

Cancerous lesions are usually detected during clinical examination of oral tissues, particularly in areas such as the tongue, alveolar ridge, gingiva, floor of the mouth, cheeks, and palate [6]. The treatment of this disease is determined by the patient’s systemic health, the carcinoma stage, and potential outcomes in terms of function, aesthetics, and quality of life [7]. Surgery is nowadays the primary treatment; however, it is linked to morbidity and limitations in functional and aesthetic activities, implying even social discomfort and stress [8]. In some cases, surgical treatment also requires postoperative complementation with radiotherapy and chemotherapy using single-agent platinum [9,10]. However, these alternatives may lead to tolerance, toxicity, and chemoresistance [10]. Despite advances in treatment, the 5-year survival rate for patients with advanced-stage OSCC remains less than 50% [11], showing the urgent need for improved treatments and early detection methods for oral cancer.

Mutations in tumor suppressor genes, oncogenes, or cell-cycle regulatory genes can lead to the development of human cancer. The role of p53 in the development of OSCC has been extensively studied over the past few decades. p53 is also regarded as a pivotal hallmark in OSCC development, exhibiting a dual functionality. The p53 gene, also known as *TP53*, is a tumor suppressor gene that encodes the tumor protein p53. This gene plays a crucial role in various signaling pathways, including the cell cycle, DNA repair, and cell death through apoptosis, autophagy and ferroptosis (Figure 1) [12]. The wild-type p53 (wt p53) regulates genes that suppress tumor formation and contribute to maintaining genomic stability. Conversely, mutations in the p53 gene (mut p53) disrupt normal gene expression [13,14,15,16], which leads to mutations in the p53 protein, which result in either a loss of function (LOF), causing an inability to activate the target genes regulated by wild-type p53 (wtp53), or gain-of-function (GOF) effects inducing the interaction of mut p53 with proteins with which wtp53 does not typically interact. The inability of p53 to activate genes in LOF affects the pathways that regulate tumor suppression; conversely, GOF effects are essential for the proliferation and survival of malignant cells. Therefore, therapeutic strategies have focused not only on restoring wild-type activity in LOF but also on inhibiting the GOF mutant protein [17].

p53 alterations are frequently identified in precancerous lesions, with a prevalence of 10% to 50% [24]. Although studies have been contradictory, some have reported alterations in the initial stages of oral carcinoma development, while others have found alterations in later stages [25,26,27]. Shin et al. observed deregulated p53 expression in advanced stages of dysplasia and OSCC [28]. Shahnavaz et al. identified genetic mutations in the p53 gene, including nonsense, splice, and missense mutations, in 75% of analyzed OSCC cases and 8% of dysplastic lesions. Their findings suggest that these mutations are associated with invasive and late-stage carcinoma phenotypes [29,30].

Although TP53 mutations are highly prevalent in OSCC, the greatest obstacle is the clinical translation of therapies targeting p53. This stems from a lack of understanding of the mechanistic relationship between these therapies and the heterogeneity of mut p53 in OSCC, as well as the fact that many drugs are still in preclinical trials and have not been approved for clinical use. Furthermore, these drugs lack selectivity for mut p53, which means they might damage healthy tissues. This review aims to provide a state-of-the-art review of the outcomes of these therapies in OSCC [4,14].

## 2. Methodology

The literature was reviewed in the PubMed and Web of Science databases using keywords such as oral squamous cell carcinoma, *TP53*, and p53 therapies, with a specific range from 2020 to 2025. Only articles addressing p53-targeted therapies in oral squamous cell carcinoma were included, excluding studies on HNSCC (epidermoid skin carcinoma, pharyngeal carcinoma, laryngeal carcinoma, etc.) that did not include oral squamous cell carcinoma. However, in some cases, general information on HNSCC was included when specific data on OSCC were limited.

## 3. Types of Therapies Directed Against p53

In recent decades, diverse strategies have been developed to restore p53 function and activity in cancers with mutations in the *TP53* gene. These strategies include gene therapy using wt p53 with adenovirus vectors and the design of oncolytic viruses that replicate and selectively eliminate cells with mut p53, along with the development of siRNA and antisense RNA that reactivate or release p53 activity by inhibiting the function of negative regulators such as Mdm2, MdmX, and HPV E6, as well as small-molecule therapy that directly or indirectly activates p53 [15,31].

## 4. p53-Targeted Therapies for Oral Squamous Cell Carcinoma

Therapies targeting p53 for OSCC fall under HNSCC therapies and were classified into three categories according to p53 status, (1) wt p53, (2) mut p53, and (3) human papillomavirus (HPV)-positive HNSCC, or according to the function of the molecule or compound, (1) binding to mut p53 to restore it to a wild-type conformational state, (2) inducing degradation or inhibition, directly or indirectly, of mut p53, (3) enhancing synthetic lethality of mut p53, (4) inhibiting protein–protein interactions, (5) targeting viral enzymes responsible for p53 degradation, and (6) activation of the immune system [32]. However, over the last five years, research has evolved towards combinations of molecules that target p53 as a primary therapeutic strategy while also activating other signaling pathways or involving p53 secondarily [15,32,33,34]. In this article, we will categorize the therapies into the following groups: therapies binding to mut p53 to induce a conformational change, p53-targeted small-molecule therapy, therapies targeting mut p53 at the genetic and protein levels, therapies targeting HPV-positive OSCC, oncology virotherapy, and other therapies.

### 4.1. Therapies Targeting to Mut p53 to Induce a Conformational Change

#### 4.1.1. 4-Hesoxyresorcinol (4HR)

4HR, a pharmacological chaperone with antioxidant properties and a histone deacetylase (HDAC) inhibitor, has shown potential as a therapeutic strategy (Table 1). Studies in the YD-15 cell line (mucoepidermoid carcinoma of the tongue with mut p53) showed that 4HR decreases cell viability and promotes apoptosis by inducing overexpression of the pro-apoptotic BAX and BAD and downregulation of Bcl-2, an anti-apoptotic protein. Furthermore, 4HR treatment increased BAX and BAD in the murine YD-15 cell xenograft model compared to the control group. Additionally, 4HR induces a conformational change in mut p53, which increases its binds to DNA and, in turn, its transcriptional activity, imitating the function of wt p53 [34]. Another study described that 4HR restores the loss of function of mut p53 in the YD-15 cell line through processes such as acetylation and phosphorylation of p53. The phosphorylation of p53 increases its stability, while acetylation improves its DNA-binding affinity, which in turn enhances the transcription of target genes [35]. These findings underscore the potential of 4HR as a promising therapeutic strategy for mucoepidermoid carcinoma of the tongue with mut p53 as well as OSCC with wt p53.

#### 4.1.2. CP-31398

CP-31398 is a chemical compound that specifically targets mut p53 by stabilizing its active form, enhancing DNA binding, and promoting transcription (Table 1). Several preclinical studies have demonstrated the significant potential of the small molecule CP-31398 in restoring the function of mut p53 in head and neck cancer. CP-31398 significantly inhibits cell growth and increases the cyclin-dependent kinase inhibitor p21 protein in mutant *TP53* HNSCC cell lines, such as UMSCC-22A (cell line derived from the floor of the mouth) and FADU, compared to wild-type *TP53* JHU-028, whereas p21 induces a cell-cycle arrest in the G1 phase by inhibiting the CycD/CDK4/6 complex [36], and treatment with CP-31398 was tested only in a preclinical trial using oral cell lines, including the squamous cell carcinoma cell lines HSC-2 (derived from human oral mucosa), HSC-3, and HSC-4 (derived from human tongue), and Ca9-22 cells (derived from human gingival squamous cell carcinoma). The antiproliferative effect was observed only in the HSC-3 cells, whereas the other cell lines showed no change during treatment [37].

### 4.2. p53-Targeted Small-Molecule Therapy

#### 4.2.1. COTI-2

COTI-2 is a third-generation thiosemicarbazone small molecule with antiproliferative activity (Table 2). It has been proposed that COTI-2 binds to misfolded mutant forms of the p53 protein, restoring its function and upregulating the p53 WT and mut p53 genes. A study performed in PCI-13 cells (retromolar trigone squamous cell carcinoma), either with null p53 expression, or expressing wt p53, or GOF mut p53, showed that COTI-2 normalizes expression of the wt *P53* gene and restores the binding of the mut p53 protein to DNA. This leads to decreased cell viability and clonogenic survival, regardless of p53 status in PCI-13 cells. Furthermore, COTI-2 induces DNA damage and replication stress, leading to apoptosis (in cell lines with null or mutant p53) via PARP hydrolysis and activation of caspases-9 and -3, and to senescence (in cell lines with wt p53) via p21 expression and β-galactosidase activity; both are associated with low survival. Additionally, COTI-2 potentiates the antitumoral response to radiation and/or cisplatin in vitro and in vivo models with mut p53 HNSCC cells [38]. In addition, COTI-2 induces the activation of the calcium-/calmodulin-dependent protein kinase kinase 2 (CaMKK2)/AMPK signaling pathway and mTOR inhibition in HNSCC cells independent of p53 status, whereas AMPK could inhibit the pro-oncogenic signal induced by mTOR via phosphorylation of both mTORC1 and mTORC2 complexes [38]. In a Phase I clinical study involving patients with ovarian, uterine, and endometrial cancer, COTI-2 was shown to be safe and well tolerated. The third part of the study includes patients with HNSCC, and COTI-2 has been tested in this cancer type (NCT02433626) [39]. However, the status of this study is unknown.

#### 4.2.2. Astilbin

Astilbin is a flavonoid present in the plants *Sacandra glabra* and *Smilax glabra* Roxb, which possesses antioxidant, anti-inflammatory, immunosuppressive, and anticancer properties [42,43] (Table 2). In Aimin Wu’s study, the effect of astilbin was determined, particularly the p53-Mdm2 cross-interaction, given that Mdm2 is a negative regulator of the p53 protein [2,44]. Using OSCC cell lines SCC90 and SCC4 (with mutated p53) treated with Astilbin with and without RITA (Mdm2 antagonist), the results showed that Astilbin in combination with RITA has a greater effect in suppressing the growth of OSCC cells in a p53-dependent manner to a higher percentage than in treatments without RITA. Astilbin binds to tumor cell DNA, causing the generation of reactive oxygen species and inducing apoptosis via mitochondrial. This process involves an increase in the levels of p53, p21 and NOXA, PARP cleavage and caspase-9 and caspase-3 activation, and downregulation of Mdm2 [30]. This study demonstrates that Astilbin represents a targeted therapy for the p53-Mdm2 pathway in OSCC. However, further in vitro and in vivo studies are required.

#### 4.2.3. PRIMA-1

Initially known as PRIMA-1, this small bioactive drug has the ability to reactivate mut p53 (Table 2). It was later modified into a methylated form known as PRIMA-1-met or APR-246 (methylene-quinuclidine (MQ)). MQ binds covalently to cysteines in mut p53 and unfolds WT p53, hence reestablishing the action of p53 [45,46,47]. This drug docks at the DNA-binding site, stabilizing its structure. Upon reactivation, the p53 protein activates apoptosis-promoting genes such as *PUMA*, *BAX*, and *NOXA* [46,48]. An in vitro assay evaluated the effect of PRIMA-1 on several OSCC cell lines, including Ca9-22, HSC-2, HSC-3, and HSC-4; however, it showed significant inhibition only in the HSC-3 cell line [37]. Jong-Lyel et al. demonstrated that PRIMA-1 significantly reduced the cell viability of the mut p53 OSCC cell lines, including FADU, JHU-029, and UMSCC-22A. PRIMA-1 promotes G2 phase cell-cycle arrest and apoptosis in the UMSCC-22A line by increasing the levels of p21, Bax, and caspase-3 activity, while downregulating Bcl_XL_. Furthermore, PRIMA-1 enhances the cytotoxic effects of chemotherapeutic agents such as cisplatin, 5-fluouracil, taxol, and erlotinib on the UMSCC-22A line, whereas the co-treatment of PRIMA-1 and cisplatin induces apoptosis in UMSCC-22A cells by increasing the p21, Bax, Puma, and NOXA mRNA levels and p53 accumulation in the nucleus. The authors suggest that restoring the p53 pathway could be a therapeutic option for patients with HNSCC, potentially reducing the dose of chemotherapeutic agents required in clinical settings, thereby minimizing potential side effects in cancer patients [36]. On the other hand, piperlongumine (an alkaloid derived from *Piper longum* L.) has been shown to induce a synergistic effect with APR-246. This combination selectively reduces cell viability and clonogenic formation, while increasing both apoptotic and autophagic cell death in UMSCC10A and FADU cells. The mechanism involves induction of p21 expression, caspase-3 and -7, and PARP activation, cleaved and DNA fragmentation, as well as LC3B-II formation. This occurs through the inhibition of GSTP1 activity, leading to the formation of ROS and DNA damage, and depletion of γ-l-glutamyl-l-cysteinyl-glycine, also known as glutathione (GSH). The overexpression of GSTP1 in UMSCC10A cells significantly reduced ROS generation and induced apoptosis and autophagy under piperlongumine and APR-246 treatment. In addition, administration of piperlongumine and APR-246 inhibited tumor growth in the UMSCC10A xenograft model in SCID mice [49]. These findings support the therapeutic efficacy of this molecule in head and neck cancers [50].

### 4.3. Therapy Targeting Mut p53 at the Genetic and Protein Levels

#### 4.3.1. Metformin

Metformin (Met) is a biguanide with widely used antihyperglycemic effects; however, studies show it exhibits antitumor effects [51] (Table 3). Under hypoxic conditions, metformin enhances OSCC radiosensitivity, increasing apoptosis and reducing cell migration through reactive oxygen species (ROS) generation. This treatment also downregulates transgelin 2, an actin-binding protein (TAGLN2), upregulates glyceraldehyde-3-phosphate dehydrogenase (GA3PDH) and reduces mut p53 levels in hypoxic and radiosensitized cells [52]. It has been shown that TAGLN2 activity is necessary for *TP53* stability and, in turn, for p53 activity under hypoxia [53]. On the other hand, Met arrests the growth of the human oral tongue squamous cell line (SCC-15) through p53. Met inhibits nerve growth factor receptor (NGFR) proteolysis, decreasing the generation of the truncated form (NGFR-N) and its intracellular domain (ICD) by inhibiting the α- and γ-secretase gene expression, thereby promoting its antitumor effect [54]. NGFR-N induces cell proliferation by binding to p53, inhibiting the transcriptional activity of p53, and, consequently, the expression of Bax, PUMA, and p21 proteins [54].

#### 4.3.2. Rapalogs

Rapalogs are first-generation inhibitors of the mechanistic target of rapamycin (mTOR), including rapamycin (RAD001) and temsirolimus (CCI-779) [61]. mTOR is a kinase that belongs to the mTORC1 complex that regulates macromolecular synthesis and cell growth and is implicated in cancer etiology (Table 3). It has been reported that p53 suppresses cell growth, aided by mTORC1; therefore, rapamycin may have an additive effect with p53 in suppressing mTORC1 [62]. Md Maksudul et al. reported that RAD001 and CCI-779 induce cell death in various HNSCC cell lines (FaDu, SCC114, HN31, PCI13-R175H, PCI13-H179Y, PCI13-F134C, and PCI13-A161S), particularly in those with mut *TP53* and HPV-negative status; this cell death occurs through autophagy rather than apoptosis. Autophagy-dependent non-apoptotic cell death (ADCD) results from ULK1 activation and mTORC1 inhibition. ADCD in *TP53* HPV-negative mut *TP53* HN31 and FaDu xenografts results from the effect of RAD001, which prevents tumor growth [63]. It has been demonstrated that mut p53 promotes tumoral progression by inhibiting autophagy through the activation of mTORC1, which inactivates ULK-1 via phosphorylation at Ser^757^ and Ser^637^. ULK-1 is a Ser-Thr kinase highly involved in initiating the autophagic process [64]. These data suggest that rapamycin and temsirolimus induce their antineoplastic effects by blocking the mTORC1 activation induced by mut p53, promoting autophagy as a cell death mechanism.

#### 4.3.3. Resveratrol

Resveratrol (Res) is a natural phytoestrogen with anticancer, antimetastatic, pro-apoptotic, and tumor chemopreventive properties [65] (Table 3). A study by Chen Mao et al. on the CAL-27 and SCC-9 cell lines showed that Res induces the translocation of p53 from the cytoplasm to the nucleus, leading to the inhibition of *SLC7A11* transcription and, in turn, activating ferroptosis-mediated cell death. The study showed reduced cell viability and colony formation, as well as migration and invasion capacity and G1-phase cell-cycle arrest induced by Res on OSCC cells. This was associated with increased iron ions, elevated levels of ROS, increased lactate dehydrogenase (LDH) release, and reduced GSH and GPX-4 protein levels. Additionally, it has been observed that Res is not toxic to normal oral keratinocytes [66]. Studies show that p53 blocks cystine uptake and sensitizes cancer cells to ferroptosis by decreasing SLC7A11 expression, reducing GSH production, and increasing ROS formation. In vitro and in vivo investigations of Res against p53 in OSCC are scarce. However, a study published in 2025 concluded that Res treatment in OSCC cells induces apoptosis via cytochrome C release from mitochondria to the cytosol, decreases anti-apoptotic proteins (Bcl-2 and Bclx) and increases pro-apoptotic proteins (Bax, Bak, caspase-9, -3 and -7 activation). Res also induces autophagy by promoting ATG5 expression and LC3B-II formation. Moreover, it has an anti-metastatic effect by stimulating E-Cadherin synthesis, downregulating MT1-MMP, MMP-2, and -9 activity, and reducing the activation of transcription factors, which promotes the epithelial–mesenchymal transition (Snail, Slug, Twist, and Zeb), as well as modulating kinases involved in the antineoplastic process, such as AKT, ERK, and p38. These results suggest that Res could be considered an effective antitumor therapy in future studies [67].

#### 4.3.4. Rumex

*Rumex dentatus* L. is a plant that contains bioactive compounds such as anthraquinones and flavonoids, with antimicrobial, antioxidant, anti-inflammatory, and cytotoxic properties [68,69] (Table 3). This extract, used alone or in combination with cisplatin, has been shown to reduce cell growth dose-dependently and significantly increase p53 protein expression. It also downregulates the expression of the anti-apoptotic protein BCL2 and ATG7, and thus synergistically increases the Sub G1 cell population, apoptosis and autophagy inhibition in HNO-97 (human tongue carcinoma cell line) compared to either cisplatin or *Rumex dentatus* L. alone. Furthermore, studies demonstrated that suppressing ATG7 expression can prevent the development of precancerous lesions and decrease resistance to certain therapies [70]. This suggests that *Rumex dentatus* L. could be considered a potential adjuvant therapy, with promising improvement in cisplatin efficacy in overcoming resistance mechanisms in tongue squamous cell carcinoma through apoptosis induction and autophagy inhibition, which can function as a survival mechanism in this neoplasia [71].

#### 4.3.5. BAY-117082

BAY-117082 is a sulfonyl derivative, a potent inhibitor of NLRP3 inflammasome (nucleotide-binding domain, leucine-rich-containing family, pyrin domain-containing-3), with anticancer, apoptotic, neuroprotective, and anti-inflammatory properties [72,73] (Table 3). Studies have reported that BAY-117082 interferes with p53; specifically, a study in the CAL-27 cell line showed that BAY-117082 increases p53 protein expression and decreases Bcl2 levels, thereby promoting cell death by apoptosis. In addition, BAY-117082 reduced cell viability in HSC-2, CAL27, and SCC-4 cells. BAY-117082 treatment at higher concentrations (10 and 30 M) in CAL-27 cells significantly decreased the expression of NLRP3, apoptosis-associated speck-like protein (ASC), caspase-1, IL-18, IL-1β, and Bcl-2, and increased the expression of Bad, Bax, and p53. In vivo studies reported that BAY-117082 (doses of 2.5 and 5 mg/kg) significantly reduced subcutaneous tumor mass and the NLRP3 inflammasome response, as well as induced apoptosis via inhibition of NF-ĸB through the downregulation of the p65 NF-ĸB subunit and upregulation of IĸBα (NF-ĸb inhibitor) [59]. It has been demonstrated that NF-ĸB induces p53 degradation, contributing to cell survival, as NF-ĸB induces the E3 ubiquitin ligase Mdm2 transcription [74]. Furthermore, BAY-117082 was tested in a CAL-27 xenograft in an in vivo assay, where a dose-dependent decrease in tongue tumor mass as well as metastasis in the lymph node, spleen, and lungs was observed via inhibition of NLRP3 inflammasome activity and of the epithelial–mesenchymal transition (EMT) process through the downregulation of NLRP3, ASC, IL-1β, IL-18, N-Cadherin, MMP-2, and MMP-9, as well as increases in E-Cadherin [59,75,76]. Also, BAY-117082 significantly reduced hypopharyngeal carcinogenesis through the inhibition of NF-ĸB [73].

#### 4.3.6. RITA

The small molecule known as RITA (reactivation of p53 and induction of tumor cell apoptosis) binds to the N-terminal region of p53, modifying its structure and restoring activity in both mutant p53 and wt p53 (Table 3). Additionally, RITA binding to p53 blocks p53-mdm2 complex formation, promoting p53 stability and, in turn, its activation [60,77]. In an in vitro assay, RITA was used to treat cells with the mutant *TP53* gene from different OSCC cell lines (Ca9-22, HSC-2, HSC-3, and HSC-4), and it was observed that this molecule has high antiproliferative activity across these cell lines and is considered a promoter of apoptotic cell death. The percentages of cell inhibition were as follows: more than 70% in Ca9-22; more than 40% in HSC-2; more than 25% in HSC-3; and approximately 40% in HSC-4. In Ca9-22, inhibition of proliferation was observed after 48 h of treatment with RITA, which mediated the phosphorylation of p53 at Ser^16^ and increased BAX expression. The authors suggest that RITA induces apoptosis in Ca9-22 cells by restoring p53 transcriptional activity [37]. RITA also significantly reduced cell growth in the FADU, UMSCC-22A, JHU-O29, and wt p53 JHU-O28 cell lines. In the FADU cell line, RITA promotes apoptosis by phosphorylating p53 and increasing p21 and Bax levels. In addition, RITA showed an apoptotic effect in combination with cisplatin compared with each agent used alone, via upregulation of p21 and Bax, as well as downregulation of Bcl_XL_. The authors suggest that RITA induces its antineoplastic effects by restoring the transcriptional transactivation function of p53 [36].

### 4.4. Therapies Targeting HPV-Positive OSCC

#### Peposertib

Peposertib is an inhibitor of the DNA-dependent kinase catalytic subunit (DNA-PKcs). In vitro and in vivo studies show that it exhibits antineoplastic activity and is a potential sensitizer and enhancer of chemotherapy and radiotherapy [78,79,80] (Table 4). The first study in patients with advanced solid tumors included two patients with HNSCC and concluded that peposertib inhibits DNA-PK activity [81]. Further, a study in several HNSCC cell lines, including the UM-SCC 14A (floor of mouth squamous cell carcinoma) cell line, showed promising results for peposertib in radiosensitizing HNSCC cells, particularly based on HPV and p53 status. Therapeutic responses may be affected because the HPV E6 oncoprotein is associated with p53 degradation, whereas HPV-negative cancer is associated with *TP53* mutations. These combined treatments reduced cell viability and proliferation as well as delayed DNA repair. Cells with altered p53 induce cell-cycle arrest in the G2/M phase and are eliminated by apoptosis, while cells with functional p53 undergo senescence [82]. In HPV+ cancer cells, wt p53 predominates. However, oncogenic proteins block its tumor suppressor activity. Quinn and colleagues reported that treatment of HPV+ cancer cells with fenofibrate, in combination with PD-1/PD-L1-targeted immunotherapies, induced the accumulation and reactivation of the p53 tumor suppressor program, thereby reprogramming the tumor immune microenvironment [83]. The clinical trial conducted in several epithelial tumors confirms that peposetin, in combination with radiotherapy, is well tolerated; however, trials are currently ongoing in patients with HNSCC (NCT04533750) [84].

### 4.5. Oncologic Virotherapy

#### 4.5.1. The Recombinant Adenoviral TP53 (Ad-p53)

The use of viral particles as an alternative strategy to eliminate oncogenic cells has been investigated. These viruses would selectively replicate within and lyse the cancer cells. The adenovirus has been evaluated as a treatment against the HNSCC cells. The adenoviral vector can infect both dividing and quiescent cancer cells, remaining as an episomal vector that produces high levels of the recombinant *TP53* gene transcript, thereby inhibiting cell proliferation and tumor growth in HNSCC cells [86]. Ta-Jein et al. demonstrated that the introduction of wt p53 into Tu-138 and Tu-177 SCCHN cells via an *Ad-p53* recombinant adenoviral vector (Ad5CMV-p53; INGN 201) decreased cell viability and promoted apoptosis via overexpression of the transcription and translation of the *TP53* gene in relation to cells infected with vector only. Furthermore, treatment with peritumoral infiltration of AdSCMV-p53 reduced tumor volume in HNSCC xenograft models [87]. In another study, the administration of adenovirus Av1-p53 to the mutant p53 JSQ-3 cell line sensitized tumor cells to radiotherapy both in vitro and in vivo [88]. Additionally, it was demonstrated that docetaxel enhanced the antitumor effect of Ad-p53 on HNSCC cell lines, including mut p53 HN12 and wt p53 HN30, through upregulating the expression of exogenous p53, leading to apoptosis via downregulation of cyclin A and cyclin B, upregulation of Bax, cleavage of caspase-3 and PARP, and phosphorylation of c-Jun at Ser^63^ [89]. Stress-induced phosphorylation of c-Jun can increase apoptosis [90]. Moreover, intratumoral injection of docetaxel also enhances the cytotoxic activity of Ad-p53 in the HN-30 xenograft model, promoting a significant reduction in tumor weight compared to animals treated with either Ad-p53 or docetaxel alone [89].

Based on these promising preclinical studies, several clinical trials evaluated the safety and antitumor efficacy of Ad-p53 in the HNSCC population. Sobol R et al. analyzed Ad-p53 as a treatment in recurrent HNSCC patients. An anticancer effect was observed using Ad-p53 doses higher than 7 × 10^10^ viral particles/cm^3^ of tumor volume, along with a positive p53 biomarker profile. Ad-p53 treatment enhances the response to immune checkpoint blockade by increasing CD8+ T cells, resulting from elevated interferon signaling and decreased TGFβ and β-catenin. Therefore, Ad-p53 in combination with immunotherapy has been studied due to the association between p53 action and immune checkpoint blockade [91]. The Phase II clinical trial NCT03544723 assessed the effectiveness of gene therapy using Ad-p53 combined with anti-PD-1, an immune checkpoint inhibitor, in patients with recurrent or metastatic tumors, including HNSCC [92]. However, there have been no updates on this trial for the past two years, and the results have yet to be published.

In a randomized Phase III clinical trial, 116 patients with recurrent HNSCC were treated with methotrexate or Ad-p53. This study showed that patients with a favorable p53 profile (either wt p53 or low mut p53 expression) responded best to Ad-p53 therapy, with a survival of 7.2 months, compared to 2.7 months in patients with an unfavorable p53 profile (high mut p53 expression). On the other hand, most patients (87%) who respond to methotrexate express high levels of mut p53. This study suggests no significant clinical benefit between the two therapies [93].

##### The Recombinant Adenoviral TP53 (rAd-p53)

The first human gene therapy using recombinant adenovirus rAd-p53 (Ad5RSV-p53) for HNSCC was approved in China and is commercially known as Gendicine^®^ [94]. Gendicine is a recombinant human adenovirus with the wt p53 gene. Once the rAd-p53 therapy is applied to tumor cells, it transfers a functional copy of the p53 gene, restoring wt p53 function [95]. This promotes the expression of tumor suppressor genes and enhances the immune response, which leads to apoptosis or cell-cycle arrest [96,97]. A randomized phase II clinical trial was conducted in patients with tongue and gum cancer. In this study, the efficacy of rAd-p53 combined with radiotherapy was evaluated in 107 patients with either tongue carcinoma (TCa) or gingival carcinoma (GCa) after surgical treatment. It was evidenced that disease recurrence was 7% for the treatment group compared to 32% in the control group, and the disease-free survival rate after three years was over 90% [98]. In another randomized, double-blind phase II clinical trial, patients with stage II and IV oral carcinoma were treated with intra-arterial infusion of rAd-p53 in combination with chemotherapy. The response rate was higher with rAd-p53 plus chemotherapy (88.6%); by contrast, it was 54.5% with gendicine alone and 51.6% with chemotherapy alone. Survival rates after 3 years were 82.9%, 60.6%, and 58.1%, respectively. These findings suggest that combined therapy of rAd-p53 with chemotherapy may be a treatment alternative in patients with advanced oral cancer [99]. Zhang WW et al. reported that several studies have established the efficacy of gendicine therapy in cancer patients by restoring p53 gene function in p53-mutant tumor cells, including oral squamous cell cancer. It was observed that combining gendicine with either radiotherapy or chemotherapy leads to more successful outcomes for treated patients. They also mention that p53 status does not determine gendicine efficacy [96].

#### 4.5.2. ONYX-015

Another viral approach is ONYX-015, an oncolytic chimeric adenovirus derived from two adenovirus species, serotypes 2 (Ad2) and 5 (Ad5), which lacks the E1B-55K gene. The E1B protein binds and inactivates p53 in the infected cells. As a result of this deletion, ONYX-015 efficiently targets p53-deficient cells without affecting p53 activity in cells that express wt p53. An in vitro study has shown that ONYX-015, also known as dl1520 or CI-1042, induces tumor cell lysis in the OSCC cell line HN-5a. Furthermore, when combined with melphalan, a chemotherapeutic drug, its efficacy was enhanced by reducing cell proliferation by up to 60% [74].

In xenograft tumors from HLaC (laryngeal cancer cell line), ONYX-015 intratumoral injection over 5 consecutive days was significantly more effective at reducing tumor growth than a single application of the same total ONYX-015 dose. The mean tumor volume was 145 ± 38 mm^3^ with multiple-dose administration versus 453 ± 130 mm^3^ with a single dose, respectively [100].

Based on these results, Khury et al. reported that co-treatment of intratumoral ONYX-015 with 5-FU/cisplatin in patients with recurrent HNSCC was more active, with 63% of patients treated showing an objective response with a 50% or greater reduction in tumor size. This tumor response to ONYX-015 in combination with 5-FU/cisplatin was superior compared to the patients with HNSCC who received the 5-FU/cisplatin combination. However, in both studies, no correlation was observed between p53 status and tumor response [101].

In a Phase II clinical study, it was found that ONYX-015 replicated only in tumor cells and induced tumor necrosis without affecting surrounding healthy tissue when injected directly into recurrent head and neck tumors. Significantly, necrosis was observed more in tumors with mut p53 compared to those with wt p53 [102].

According to clinical trial results, the rAd-p53 combination therapy appears promising for tumor response and survival. The efficacy of Ad-p53 and ONYX-015 is also observed; however, further studies are needed to support these findings [91,98,103].

### 4.6. Other Therapies

#### 4.6.1. Nanoparticles

Nanotechnology has emerged as a novel therapeutic strategy in oral cancer, particularly in loading plant compounds with anticancer properties (Table 5). In this context, Tayyeb et al. designed multifunctional curcumin-mediated zinc oxide nanoparticles (ZnO NPs) that showed anticancer activity through upregulating genes such as *BAX*, *BCL2*, and *P53* in Human Oral Epidermal Carcinoma KB cells [104]. Curcumin also promotes cell-cycle arrest and apoptosis in HNSCC by activating ATM/Chk2/p53-dependent signaling in vitro and in vivo [105]. Similarly, Shaik et al. synthesized piperine-coated zinc oxide nanoparticles (ZnO-PIP NPs) that reduced cell viability and increased mRNA expression levels of BCL-2, BAX, and P53 in KB oral squamous carcinoma cells [106]. In another study, a nanoemulsion containing 5-FU and curcumin was designed; this combination decreases cell viability in squamous cell carcinoma cells, accompanied by an increase in p53- and p21-dependent apoptosis [107].

Another quinacrine–gold hybrid nanoparticle (QAuNP) also showed antiproliferative, anti-angiogenic, and antimetastatic effects on oral cancer stem cells (CSCs) via p53/p21 signaling. In vitro studies showed that QAuNPs reduced cellular proliferation and induced apoptosis in OSCC-CSCs compared with quinacrine or gold nanoparticles alone. This effect is due to decreasing mitochondrial membrane potential and Bcl-xl levels, while elevating Bax, caspase-3 activation, cleaved PARP, ROS, and NO [108]. Furthermore, QAuNP induced anti-inflammatory and anti-angiogenic effects by downregulating the expression of TNF-α, IL-1β, IL-6, IL-8, Ang-1, Ang-2, VEGF, and MMP-2, and upregulating the expression of p53 and p21. In addition, QAuNP treatment reduced tumor volume and induced apoptosis in xenograft mice by increasing the levels of p53, p21, Bax, and cleaved PARP, and by depleting Bcl-xl, Ang-1, and Ang-2. On the other hand, in H-357-PEMTp53^−/−^ p21^−/−^ cells, QAuNP treatment did not exhibit any antiproliferative, pro-apoptotic, anti-inflammatory, or anti-angiogenic effects. This suggests that the QAuNP induces its antineoplastic effect via p53 and p21. Additionally, Yang et al. demonstrated that AuNPs loaded with extracellular vesicles (EVs; abundant with ncRNAs and chrysin) called Au-EVs could stimulate apoptosis and decrease migration in SCC9 tongue squamous cell carcinoma (TSCC) in vitro, whereas apoptosis is enhanced with the co-treatment of Au-EVs with near-infrared radiation (NIR) via upregulation of let-7a-3p/p53 signaling [109].

**Table 5:** Other therapies.

Therapy	Function	Mechanism of Action	Reference
**Nanoparticles**	Inhibition of mut p53	Degrades p53 and increases the endoplasmic reticulum stress	[110]
**MEG-3**	Stabilizes p53	Interacts with the p53 DNA-binding domain for gene transcription	[111]
**MK-1775**	Sensibilization of tumor cells deficient of p53	Not established yet	[112]

#### 4.6.2. Long Non-Coding RNA: MEG3

Long non-coding RNAs (IncRNAs) have been shown to play an important role in HNSCC carcinogenesis. They are involved in regulating the p53 pathway and processes such as epithelial–mesenchymal transition and immune response, making them potential therapeutic targets [113]. Maternally expressed 3 (MEG3) is an abnormally expressed long non-coding RNA (lncRNA) and has been shown in vitro to have an apoptotic effect on the SCC25 and CAL27 cell lines of OSCC due to its effect on p53 (MEG3/p53) (Table 5). lncRNA MEG3 prevents the proliferation, migration, and invasion of SCC25 and CAL27 cells because of programmed cell death [114]. In addition, Ji et al. showed that MEG3 overexpression suppresses proliferation, EMT, migration, and invasion in HNSCC by regulating E-Cadherin through downregulation of miR-421 [115]. Furthermore, MEG3 expression reduces the self-renewal and invasive capacity of CSCs in both GNM (gingival carcinoma neck metastasis) and SAS (tongue squamous cell carcinoma) via its binding and inactivation of miR-21 [116]. It has been demonstrated that miR-421 negatively regulates the ATM/Chk2/p53 pathway, an important participant in modulating cell-cycle arrest, apoptosis, angiogenesis, invasion, and drug resistance in tumors [105,117].

#### 4.6.3. MK-1775

Although mut p53 is not the target in the assay performed by Al-Jamaei AH et al., p53 mutation is explored in an in vitro study of MK-1775 (also known as AZD-1775 or adavosertib), which is a WEE1 kinase inhibitor (Table 5). The study used tongue squamous cell carcinoma cell lines: Cal-27, SCC-25, SCC-15 with mut p53, and UM-SCC-47 with wt p53. The results showed that MK-1775 treatment increased the sensitivity of these cells to radiation, as inhibiting WEE1 forces tumor cells into mitosis and leads to cell death, especially in those with p53 alterations that depend on G2, which is regulated by WEE1 [118]. Additionally, co-treatment with RCS (HDAC6 inhibitor) and adavosertib in mut p53 HNSCC cells synergistically increased apoptotic cell death associated with aberrant mitosis. This occurs by inhibiting the phosphorylation of Chk1 at Ser^345^ and, in turn, increases the activity of CDK1 and the expression of γ-H2A.X (marker of DNA double-strand breaks). There was also an increase in caspase-3 activity and PARP cleavage in a mut p53-dependent manner [119]. Furthermore, co-administration of B02 (Rad51 recombinase inhibitor) and AZD1775 synergistically inhibited tumor growth and increased the survival of mice with HPV-positive UM SCC-47 oral tongue tumors, compared with single-treatment groups and untreated mice. This combination impairs the DNA repair process (homologous recombination) mediated by Rad51, promoting DNA damage, replication stress, and apoptosis through a decrease in the levels of phosphorylation at Tyr^15^ CDK1 [120]. Diab et al. reported that the HPV16 E6/E7 oncoproteins sensitize UM-SCC47 cells to AZD1775 through the activation of the FOXM transcriptional factor by phosphorylation at Thr^600^ via the CDK1 circuit. This activation induces the expression of mitotic genes, leading to premature entry into mitosis, through E6 activities above p53 inactivation [121].

#### 4.6.4. MAGEA4-AS1

A recent study has reported that MAGEA4-AS1 was upregulated in OSCC samples and promotes proliferation, migration, and invasion in the oral cancer cell lines HSC3 and CAL27. Mechanistically, p53 interacts with MAGEA4-AS1, forming MAGEA4-AS1-p53 complexes. This complex induces the transcription of MK2 (a transcriptional factor) by binding to the MK2 promoter region, which enhances the activation of downstream signaling pathways, such as MAPKs and PI3K/AKT, and, consequently, the epithelial–mesenchymal transition (EMT), proliferation, angiogenesis, invasion, and metastasis in OSCC cells. The authors suggest that MAGEA4-AS1 could be used as a diagnostic marker and therapeutic target for OSCC patients [122].

#### 4.6.5. ROR

LncRNA-ROR is highly associated with the progression of nasopharyngeal carcinoma (NPC). Its downregulation in CNE2 NPC cells inhibits cell proliferation by deregulating the cell cycle. This increases DNA content in the G1 phase (from 59.91% to 70.11%) but decreases it in the S and G2 phases (from 40.01% to 29.89%) by downregulating PCNA and Cyclin A. In addition, lncRNA-ROR depletion in CNE2 cells promotes apoptosis and inhibits migration and invasion by inhibiting EMT via E-Cadherin re-expression and decreases in N-Cadherin, vimentin, ZEB-1, and -2. Furthermore, the down-expression of lncRNA-ROR suppresses the chemoresistance of CNE2 cells to cis-Dichlorodiamineplatinum (II) by increasing the p53/P21 signaling pathway. These results suggest that lncRNA-ROR negatively regulates p53 [123].

#### 4.6.6. H19

H19 is overexpressed in the oral cancer cell lines FADU, UMSCC-1, CAL27, and HSC-2, as well as in oral cancer-associated fibroblasts (CAFs) [124,125]. The H19/miR-675-5p axis has been shown to inhibit p53 stability, promoting increased cell proliferation in cancer cells [126]. It has been demonstrated that miR-675-5p is a derivative of H19 and induces proliferative phenotypes, migratory ability, and a glycolytic state in oral CAFs by upregulating 6-phosphofructo-2-kinase/fructose-2,6-biphosphatase 3 (PFKFB3), leading to an increase in lactate dehydrogenase (LDH) activity and, in turn, elevated lactate levels. Importantly, the genetic inhibition of H19 in oral CAFs inhibits tumor growth in vivo, showing that H19 induced by oral CAFs was correlated with the capacity of oral cancer cells to promote tumor growth by reducing E-cadherin (EMT marker) as well as increasing Ki-67 (proliferative marker) and PFKFB3 levels [125]. Lactate released by CAFs is an oncometabolite that is uptaken by tumor cells through the monocarboxylate transporter 1 (MCT1), which is transformed by LDH to pyruvate, producing large amounts of ATP through oxidation in the tricarboxylic acid (TCA) cycle [127].

## 5. Discussion

The p53 gene plays a key role in the development of various malignant tumors, as it is involved in cellular mechanisms that regulate cell proliferation and cell death. Numerous investigations in OSCC have shown that p53 overexpression and/or mutation are crucial for the development of this disease [128,129,130]. As a result, p53 remains a therapeutic target, and in recent decades, efforts to develop alternative therapeutics targeting p53 have continued.

Although therapies aimed at restoring or modulating p53 in cancer treatment have shown favorable effects, obstacles have limited their use and progression to more advanced clinical trials [131] (Table 6). Barriers to these therapies include administration, efficacy, stability, selectivity, and off-target toxicity [132]. For small molecules such as PRIMA, COTI-2, astilbin, and MDM2, a lack of selectivity has been observed, leading to off-target cytotoxicity [133,134]. Viral vectors can induce strong immunogenicity, which may either hinder or enhance therapy [135].

Another obstacle is intratumoral heterogeneity driven by different mutations at p53 gene hotspots, which generates tumor subclones that respond unevenly to p53-reactivating drugs and compromise therapeutic efficacy [136]. Specifically, mutations such as R175H (structural mutation), R248Q (contact mutation), and R273H (contact mutation) remodel the central DNA-binding domain of p53, resulting in a neomorphic GOF that confers therapeutic sensitivity [137].

Despite the challenges observed in studies of p53-targeted therapies for OSCC, this area represents an opportunity in precision oncology. Currently, the molecular synergy between targeted therapies and conventional treatments such as chemotherapy, radiotherapy, and immunotherapy offers a promising therapeutic alternative. The integration of these therapeutic combinations targets various mechanisms, including inhibition of checkpoints, sensitizing of cells to DNA damage, and restoring apoptosis, among others, thereby modifying tumor behavior and improving the therapeutic response [32,131,138].

## 6. Conclusions

In recent years, the development of p53-targeted therapies has led to a personalized approach to improve OSCC treatment based on p53 status (wt or mutant) and HPV positivity. Despite this, the status of p53 is unclear in some research. Since p53 plays a crucial role in multiple mechanisms, regulates other genes and proteins, and is itself regulated by them, it represents a potential therapeutic target in oral cancer. Further studies are needed to elucidate the mechanisms of action of these therapies with the most significant potential for treating this pathology and to continue clinical research.

## Figures and Tables

**Figure 1:**
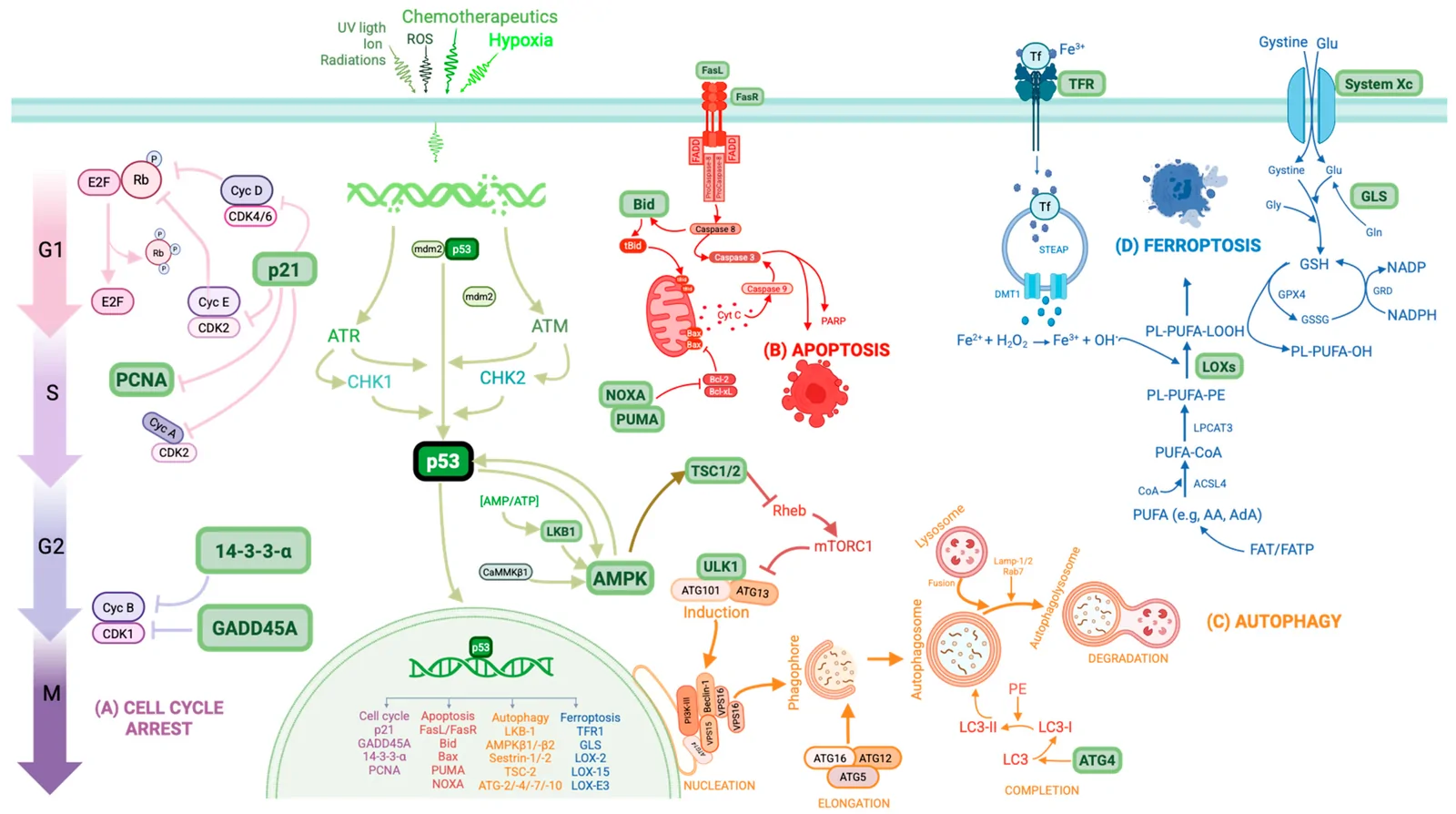
Modulation of DNA damage response, cell-cycle arrest, apoptosis, autophagy, and ferroptosis by p53. DNA damage, such as DSBs and ssDNA, activates the ATM/CHK2 and ATR/CHK1 pathways and, in turn, stabilizes p53. p53 induces genes that modulate DNA damage repair, cell-cycle arrest, apoptosis, autophagy, and ferroptosis. (**A**) Cell-cycle arrest (purple): The cell cycle consists of four phases—G1, S, G2, and M—and is regulated by CDKs, CDKIs, and phosphorylation events. P53 promotes G1/S arrest mainly by inducing p21, which inhibits cyclin D/CDK4/6 and cyclin E/A-CDK2 complexes, preventing Rb hyperphosphorylation and E2F-mediated transcription of S-phase genes. p53 also induces GADD45A, which binds CDK1 and disrupts the cyclin B/CDK1 complex, and 14-3-3σ, which inhibits cyclin B/CDK1 formation, promoting G2/M arrest. (**B**) Apoptosis (red): The apoptosis pathway is activated when FasL binds death receptors, activating FADD and the initiator caspases 8 and 10; these, in turn, activate the effector caspases 3 and 7. Caspase-8 cleaves Bid into tBid, which triggers Bax translocation to the mitochondria and releases cytochrome c. Cytochrome c activates Apaf-1 and caspase-9, which in turn promotes the activation of other effector caspases, such as caspase-3 and caspase-7. Bcl-2 and Bcl-XL are anti-apoptotic proteins that inhibit the release of cytochrome c. p53 promotes apoptosis by inducing FASL, FAS, Bid, Bax, Puma, and Noxa, while directly inhibiting Bcl-2/Bcl-XL to favor Bax/Bak-mediated mitochondrial apoptosis. (**C**) Autophagy (orange): Autophagy is a cellular process by which the cell breaks down and recycles cellular components through the formation of autophagosomes, which, upon fusing with lysosomes, facilitate the breakdown and recycling of these cellular components. Under conditions of nutrient abundance, mTORC1 suppresses autophagy, whereas stress or nutrient deprivation activates AMPK, inhibits mTORC1, and promotes autophagy. Diverse proteins that modulate this process include ATG, Beclin-1, LC3, Lamp-1/-2, and Rab7. The p53 protein promotes autophagy by regulating LKB-1, AMPK, Sestrin-1/-2, TSC-2, ULK1, and Atg-2, -4, -7, and -10. (**D**) Ferroptosis (blue): Ferroptosis is an iron-dependent form of non-apoptotic cell death that is characterized by the accumulation of lipid hydroperoxides. Transferrin-bound Fe^3+^ enters cells through transferrin receptors (TFRs), is converted to Fe^2+^ by STEAP, and enters the labile iron reservoir via DMT1 and drives Fenton reactions to generate lipid peroxides and hydroxyl radicals, thereby promoting ferroptosis. PUFAs, including arachidonic and adrenic acids, are incorporated into phospholipids by ACSL4 and LPCAT3 and subsequently oxidized by lipoxygenases (LOX-12/-15/-16 and LOXE3). P53 can induce ferroptosis by inhibiting the expression of SLC7A11 (a component of the Xc- system) and inducing the transcription of TFR1, GLS, LOX-2, LOX-15, and LOXE3. The red arrow indicates inhibition, and the green arrow represents the promoting effect. This figure was designed using data from Liu et al. (2020); Rahman et al. (2022); Aubrey et al. (2018); Park et al. (2017); Chehab et al. (2000) and Szabó (1979) [18,19,20,21,22,23]. Created with Biorender.com.

**Table 1:** Therapies targeting mut p53 to induce a conformational change.

Therapy	Function	Mechanism of Action	Reference
**4-hesoxyresorcinol**	Conformational changes and loss of function restoration	As molecular chaperone, 4HR binds to the mutated loci of the aberrant form, inducing a protein structure similar to the native one and replacing the previously misfolded structure.	[34]
**CP-31398**	Restoring p53 function	Stabilizes the core structure of p53 and inhibits ubiquitination	[36]

**Table 2:** p53-targeted small-molecule therapy.

Therapy	Function	Mechanism of Action	Reference
**COTI-2**	Restores p53 function	A zinc metal chaperone that restores zinc ions in mut p53 protein	[40]
**Astilbin**	Induces p53 expression	It is established that Astilbin downregulated MDM-2, but it is not known how	[30]
**PRIMA-1**	Reactivates p53	PRIMA-1 in vivo is converted to 2-methylene 3-quinuclidinone (MQ), a nucleophile acceptor that reacts with cysteine and thiol groups, restoring p53 conformation	[41]

**Table 3:** Therapy targeting mut p53 at the genetic and protein levels.

Therapy	Function	Mechanism of Action	Reference
**Metformin**	Decreases the levels of mut p53	SIRT-1 is upregulated by Metformin, then deacetylation of p53 mutant induces gene silencing	[55]
**Rapalogs**	Inhibit p53 signal downstream	Preventing p53 signal downstream by avoiding assembly of mTORC molecules recently synthesized	[56]
**Resveratrol**	Probably target of p53	Downregulation through MDM-2 or SIRT-1 activation	[57]
**Rumer**	Could induce increment in p53 protein	Not evaluated yet	[58]
**BAY-117082**	Increases p53 protein levels	Probably by MDM-2 regulation, more studies are required	[59]
**RITA**	Reactivation of p53	Binds to p53 and avoids its interaction with MDM-2	[60]

**Table 4:** Therapies targeting HPV-positive OSCC.

Therapy	Function	Mechanism of Action	Reference
**Peposertib**	P53 protein increment	Activates ATM/p53 response	[85]

**Table 6:** Therapies against p53 in OSCC.

Therapy	Nature of Therapeutic Agent	Type of Study (Cell Line or Human Cancer or Clinical)	IC50/ % Cell Viability	Cell Death Pathway Activated	p53 Status/Mechanism of Action	Reference
**4-hesoxyresorcinol (4HR)**	Current chemical drug Limitation: secondary toxicological effects, under study	In vitro (YD-15 and YD-9 tongue cancer cell) In vivo (xenograft of YD-15 cells in athymic nude male mice)	1–100 μM 24 h 48 h 90% 90–50% 90–80% 70–40% 10 mg/kg Differences with the control group was not significant	Intrinsic pathway of apoptosis	YD-15 (Glu258Ala) YD-9 (WT) 4HR changed the conformation of mut peptide of p53, making it similar to wild-type and increasing transcriptional activity	[34]
**Rapalogs**	Current chemical drug Limitations: drug resistance, bioavailability, under study	In vitro (SCC114 and PCI13 human oral squamous cell line) In vivo (HN31 and FaDu cell lines on tumor cell xenograft model in nude mice)	RAD001 1–1000 nM ±70–45% ±60–00% ±55–50% ±70–60% ±70–50% CCI-779 1–1000 ng ±75–65% ±60–50% ±75–60% ±70–70% ±65–55% RAD001 5 mg/kg Tumor volume reduction	Apoptosis and ferroptosis	SCC114(Arg248Gln) PCI13 (Arg175His) PCI13 (HIs179Tyr) PCI13 (Phe134Cys) PCI13 (Ala1615) SCC114(Arg248Gln) PCI13 (Arg175His) PCI13 (His179Tyr) PCI13 (Phe134Cys) PCI13 (Ala1615) HN31 (Cys176Phe) FaDu (Arg248Leu) Rapalogs promote autophagy-dependent cell death in HPV-negative mutTP53 via the ULK1 pathway	[63]
**COTI-2**	Current chemical drug Limitations: cytotoxicity independent of p53 status, under study	In vitro (human oral squamous cell line) In vivo (PCI13 cell lines xenografted on tongues of male athymic nude mice)	8.0 nmol/L 10.6 nmol/L 10–5 nmol/L 8.7 nmol/L 1.4 nmol/L 1.4 nmol/L 75 mg/kg Reduced tumor growth in combination with cisplatin	Apoptosis	PCI-13 (WT) PCI-13(Gly245Asp) PCI-13 (Cys238Phe) PCI-13 (Arg175His) PCI-13 (Arg273His) PCI-13 (Arg282Trp) PCI13 (Gly245Asp) COTI-2 restores site specific-DNA binding properties of mut p53 protein	[38]
**Peposertib**	Current chemical drug Limitations: dual mechanism of action, under study	In vitro (UM-SCC-14A squamous cell carcinoma) In vivo (xenograft models with UD-SCC-2 cell line HPV-positive: p53 inactivated)	300 nmol + 4 Gy 200 mg	Apoptosis	UM-SCC-14A (Mutant p53) (Arg273His) Peposertib radiosensitizes (by inhibiting enzymes in DNA repair pathway) head and neck squamous cell carcinoma with a better response in models with mut p53.	[82]
**rAd-p53**	Adenovirus Limitations: neutralizing antibodies generated by the immune system or poor tumor penetration, under study	Clinical (tongue cancer or gingival carcinoma) Clinical (oral carcinoma, patients on stage 3 or 4)	1 × 10^12^ viral particles with radiotherapy 3-year disease-free survival was 93% Dose 1–4 × 10^12^ viral particles with chemotherapy 3-year overall survival was 60–83%	Apoptosis	TCa and GCa P53 mutation is not indicated stage III or IV oral carcinoma P53 mutation is not indicated rAd-p53, gene therapy with a vector for transference of a wild-type p53 in cells with p53 mutated or inactivated	[98,99]
**Ad-p53**	Adenovirus Limitations: neutralizing antibodies generated by the immune system or poor tumor penetration, under study	Clinical (head and neck squamous cell carcinoma patients)	Dose greater than 7 × 10^10^ viral particles/cm^3^ Less than 20% of p53 truncations, deletions, or frame-shift mutations	Apoptosis	Patient with recurrent HNSCC P53 was genotyped, but no mutations were indicated. Ad-p53, gene therapy with a vector for transference of a wild-type p53 in cells with p53 mutated or inactivated	[91]

## Data Availability

No new data were created or analyzed in this study. Data sharing is not applicable to this article.

## Abbreviations

The following abbreviations are used in this manuscript:

4HR	4-hesoxyresorcinol
ADCD	Autophagy-dependent non-apoptotic cell death
Ad	Adenoviral
CaMkk2	Calmodulin-dependent protein kinase kinase 2
CCI-779	Temirolimus
CDKs	Cyclin-dependent kinases
CSCs	Cancer stem cells
DNA-PKcs	DNA-dependent kinase catalytic subunit
EMT	Epithelial–mesenchymal transition
G1	Growth
G2	Preparation for mitosis
GAPDH	Glyceraldehyde 3-phosphate dehydrogenase
GCa	Gingival carcinoma
GNM	Gingival carcinoma neck metastasis
GSH	Tripeptide, γ-l-glutamyl-l-cysteinyl-glycine known as glutathione
HDAC	Histone deacetylase
HLaC	Laryngeal cancer cell line
HNSCC	Head and neck squamous cell carcinoma
HPV E6	Human papillomavirus viral protein 6 encoded
ICD	Intracellular domain
IncRNAs	Long non-coding RNAs
LDH	Lactate dehydrogenase
LKB-1	Liver kinase B1
M	Mitosis
Mdm2	Murine doble minute 2
MdmX	Murine Double Minute X
MEG3	Maternally expressed 3
Met	Metformin
MQ	Methylene-quinuclidine
mTOR	The mammalian target of rapamycin
mTORC1	Mammalian target of rapamycin complex 1
NGFR	Nerve growth factor receptor
NLRP3	Nucleotide-binding domain, leucine-rich-containing family, pyrin domain containing-3
OSCC	Oral squamous cell carcinoma
PUFA	Polyunsaturated fatty acids
QAuNP	Quinacrine–gold hybrid nanoparticle
RAD001	Rapamycin drug
rAd-p53	Recombinant adenovirus-p53
Res	Resveratrol
RITA	Reactivating p53 and Inducing Tumor Apoptosis
ROS	Reactive oxygen species
S	Synthesis
SAS	Tongue squamous cell carcinoma
TAGLN2	Transgelin-2
TCa	Tongue carcinoma
TK-1	Thymidine kinase
ULK1	UNC-51-like kinase 1
Wtp53	Wild-type p53
ZnO NPs	Zinc oxide nanoparticles
ZnO-PIP NPs	Piperine-coated zinc oxide nanoparticles
